# Metabolic and Muscular Determinants of Weaning Failure: The Role of BUN/Creatinine Ratio and Rectus Femoris Thickness

**DOI:** 10.3390/jcm15010314

**Published:** 2026-01-01

**Authors:** Erdem Yalçınkaya, Muhammet Topçu, Umut Sabri Kasapoğlu, Hüseyin Arıkan, Hasan Basri Yapıcı, Semiha Emel Eryüksel, Sait Karakurt

**Affiliations:** 1 Department of Pulmonary and Critical Care Medicine, Marmara University School of Medicine, 34854 Istanbul, Türkiye; drmuhammettopcu@gmail.com (M.T.); umutkasapoglu@gmail.com (U.S.K.); arikanhuseyin@gmail.com (H.A.); dreryuksel@gmail.com (S.E.E.); saitkarakurt@hotmail.com (S.K.); 2 Department of Internal Medicine, Marmara University School of Medicine, 34854 Istanbul, Türkiye; hbasriyapici@gmail.com

**Keywords:** weaning failure, BUN/Creatinine ratio, catabolism, ICU-acquired sarcopenia, muscle ultrasound, mechanical ventilation, rectus femoris

## Abstract

**Background**: Weaning failure remains a major challenge in intensive care practice, often reflecting the interplay between systemic catabolism and skeletal muscle wasting. The blood urea nitrogen-to-creatinine (BUN/Cr) ratio is a routinely available biochemical index influenced by renal handling, hemodynamic status, protein metabolism, and muscle mass, and has been associated with adverse outcomes in critical illness. This study aimed to evaluate the association between BUN/Cr ratio, weaning outcomes, and ultrasound-based rectus femoris thickness. **Methods**: This retrospective observational study included 42 mechanically ventilated adults admitted to the medical ICU of Marmara University between December 2024 and September 2025. Rectus femoris thickness was measured via bedside ultrasonography at the time of the spontaneous breathing trial (SBT). Weaning success was defined as extubation without reintubation, death, or need for NIV/HFNO due to respiratory distress within 7 days. Laboratory and clinical variables—including BUN/Cr ratio, SOFA, APACHE II, mNUTRIC, and albumin—were recorded. Multivariable logistic regression and receiver operating characteristic (ROC) analyses were performed. **Results**: Weaning failure occurred in 13 patients (31.0%). These patients had higher BUN/Cr ratios (58.7 [44.6–76.9] vs. 39.7 [23.8–49.2], *p* = 0.007) and lower rectus femoris thickness (6.2 [5.4–7.0] vs. 7.8 [6.9–8.6] mm, *p* = 0.021). The BUN/Cr ratio independently predicted weaning failure (OR 1.07; 95% CI 1.01–1.14; *p* = 0.024). ROC analysis identified a BUN/Cr cut-off of 44.6 (AUC = 0.76) for weaning failure. An exploratory composite metabolic–muscle indicator (MMI), combining BUN/Cr ratio and rectus femoris thickness, demonstrated higher discriminative performance in this cohort (AUC = 0.81). **Conclusions**: An elevated BUN/Cr ratio was independently associated with weaning failure and lower rectus femoris thickness in this cohort. Given the observational design and potential confounding, these findings should be interpreted as hypothesis-generating. Combined biochemical and ultrasound-based assessment highlights the potential value of integrating metabolic and morphologic information when characterizing patients at risk for weaning failure. However, whether incorporation of such markers into clinical decision-making improves weaning outcomes requires prospective validation.

## 1. Introduction

Weaning from mechanical ventilation (MV) represents a pivotal and often challenging stage in the management of critically ill patients. Despite advances in ventilatory support and weaning protocols, approximately 20–30% of patients experience weaning failure, leading to prolonged mechanical ventilation, extended ICU stay, and increased mortality [1].

Successful liberation from the ventilator requires the coordinated recovery of respiratory mechanics, cardiovascular stability, metabolic balance, and skeletal muscle function [2]. Hence, understanding the physiological and biochemical factors that influence this process is fundamental to improving patient outcomes.

In critically ill patients, catabolic metabolism and skeletal muscle wasting—collectively described as ICU-acquired sarcopenia (ICU-AS)—are among the most important determinants of poor recovery and delayed weaning [3]. This condition arises due to systemic inflammation, immobilization, inadequate nutrition, endocrine dysregulation, and oxidative stress, resulting in a rapid decline in muscle mass and strength [4].

Bedside ultrasound evaluation of rectus femoris (RF) muscle thickness has emerged as a reliable, non-invasive, and reproducible tool for quantifying skeletal muscle depletion [5]. Previous studies demonstrated that reduced RF thickness correlates with longer weaning duration, extubation failure, and higher mortality [6].

Alongside these structural changes, biochemical markers of catabolic stress can provide valuable insight into the metabolic state of critically ill patients. Among them, the blood urea nitrogen to creatinine ratio (BUN/Cr) is a simple, universally available laboratory parameter reflecting nitrogen metabolism, renal perfusion, and systemic catabolism [7].

While traditionally interpreted as a marker of prerenal azotemia, emerging evidence suggests that in ICU patients, BUN/Cr elevation primarily represents increased protein breakdown and decreased creatinine generation due to muscle loss [8].

Recent studies have associated high BUN/Cr ratios with poor outcomes in sepsis, acute respiratory distress syndrome (ARDS), heart failure, and critical illness [3,6]. This relationship likely reflects the dual impact of hypercatabolism and skeletal muscle depletion on physiological reserve.

Given these converging pathways, the BUN/Cr ratio may serve as a biochemical correlate of sarcopenia, integrating metabolic and muscular dimensions of critical illness. However, despite its potential clinical utility, data on the relationship between BUN/Cr, skeletal muscle thickness, and weaning outcomes remain limited and heterogeneous.

The present retrospective study was therefore designed to investigate whether an elevated BUN/Cr ratio is associated with weaning failure and reduced rectus femoris thickness as a marker of ICU-acquired sarcopenia in mechanically ventilated patients.

We hypothesized that patients with a higher BUN/Cr ratio—reflecting systemic catabolic stress—would exhibit lower muscle mass and poorer weaning outcomes.

## 2. Materials and Methods

### 2.1. Study Design and Population

This retrospective observational study was conducted in the Medical Intensive Care Unit (ICU) of Marmara University School of Medicine between February 2025 and September 2025.

All adult patients (≥18 years) who received invasive mechanical ventilation (IMV) for ≥48 h and underwent at least one spontaneous breathing trial (SBT) were screened for eligibility.

Patients were excluded if they had pre-existing neuromuscular disease, end-stage renal disease on chronic dialysis, tracheostomy at the time of evaluation, history of recent intubation (<6 months), continuous neuromuscular blockade, or incomplete ultrasound/laboratory data.

### 2.2. Weaning and Extubation Protocol

Daily SBTs were performed when patients met established readiness criteria (1–3):

- Fraction of inspired oxygen (FiO_2_) ≤ 0.5
- Positive end-expiratory pressure (PEEP) ≤ 5 cmH_2_O
- Norepinephrine ≤ 0.2 µg/kg/min
- Rapid shallow breathing index (RSBI) < 105

SBTs were performed either using pressure support ventilation (PS 7 cmH_2_O, PEEP 5 cmH_2_O) or a T-piece trial for 30 min.

Patients tolerating the trial were extubated and followed for 7 days post-extubation.

### 2.3. Definition of Weaning Outcomes

- **Weaning success**: Extubation without reintubation, death, or need for noninvasive ventilation (NIV) or high-flow nasal oxygen (HFNO) due to respiratory distress within 7 days, consistent with international consensus [1].
- **Weaning failure**: Reintubation, death, or need for NIV/HFNO within 7 days due to respiratory distress.

### 2.4. Ultrasound Assessment of Rectus Femoris Thickness

Bedside ultrasonography was performed using an Esaote MyLab™ Seven ultrasound system (Esaote S.p.A., Genoa, Italy) equipped with a 5–12 MHz linear transducer, following standardized methodology [9,10,11].

Patients were placed supine with legs extended and relaxed.

A line was drawn from the anterior inferior iliac spine (AIIS) to the superior border of the patella; the midpoint was identified as the measurement site.

The probe was positioned perpendicular to the skin in B-mode, and the distance between superficial and deep fascia of the rectus femoris was measured.

Each measurement was repeated three times per leg, and the mean of six readings was recorded.

All ultrasound measurements were performed by a single intensivist with formal certification and more than five years of experience in critical care ultrasonography, using a standardized acquisition protocol to minimize measurement variability.

### 2.5. Definition of ICU-Acquired Sarcopenia

Low rectus femoris muscle thickness was operationally defined as values below the sex-specific 25th percentile of the study population, serving as a cohort-relative indicator of reduced muscle mass rather than a diagnostic definition of sarcopenia [12].

Patients were categorized as sarcopenic or non-sarcopenic accordingly.

### 2.6. Laboratory Parameters

For each patient, BUN and serum creatinine levels obtained within 24 h prior to the SBT were recorded, and the BUN/Cr ratio was calculated.

Additional laboratory variables included serum albumin, total protein, and inflammatory markers (e.g., CRP, leukocyte count).

These data were used to assess nutritional and catabolic status.

### 2.7. Data Collection

Demographic, clinical, and physiological data were extracted from the electronic ICU database, including:

- Age, sex, body mass index (BMI)
- Comorbidities
- APACHE II and SOFA scores (at admission and weaning)
- mNUTRIC score and Charlson Comorbidity Index
- Frailty score
- ICU length of stay and 28-day mortality
- Post-extubation need for NIV or HFNO
- BUN/Cr ratio and rectus femoris thickness.

### 2.8. Sample Size

A total of 42 patients met the inclusion criteria and were included in the final analysis.

Given the exploratory retrospective design and limited event counts, a formal a priori power analysis was not performed, and the study was not powered for definitive multivariable inference.

### 2.9. Statistical Analysis

Statistical analyses were performed using R version 4.4.2.

Continuous variables were tested for normality using the Shapiro–Wilk test and expressed as mean ± standard deviation (SD) or median [interquartile range, IQR] as appropriate.

Comparisons between groups were made using the Student’s *t*-test or Wilcoxon rank-sum test.

Categorical variables were compared with the chi-square test or Fisher’s exact test when expected counts were <5.

Correlations between continuous variables (e.g., BUN/Cr, albumin, RF thickness, mNUTRIC) were assessed using Pearson’s or Spearman’s correlation coefficients.

A multivariable logistic regression model was constructed to identify independent predictors of weaning failure, including BUN/Cr, rectus femoris thickness, SOFA, mNUTRIC, and albumin.

Results were reported as odds ratios (OR) with 95% confidence intervals (CI).

ROC curve analyses were performed to evaluate discriminative performance for weaning failure and 28-day mortality, with the optimal cut-off identified using Youden’s index [13].

A *p*-value < 0.05 was considered statistically significant.

### 2.10. Ethical Approval

This study was approved by the Marmara UniversitySchool of Medicine Clinical Research Ethics Committee (Decision No.: 09.2025.25-0882 Date: 17 October 2025)

The study was conducted in accordance with the Declaration of Helsinki.

### 2.11. Use of Generative AI

Generative artificial intelligence (GenAI) tools were used solely to assist with language editing and grammatical refinement of the manuscript. No GenAI tools were used for study design, data collection, data analysis, data interpretation, figure generation, or reference selection. All scientific content, analyses, and conclusions were developed and verified by the authors.

## 3. Results

A total of 42 mechanically ventilated patients were included in the final analysis. Among them, 29 (69.0%) were successfully weaned, while 13 (31.0%) experienced weaning failure. Baseline demographic, clinical, and laboratory characteristics of both groups are summarized in Table 1.

Among the 13 patients classified as having weaning failure, 4 required reintubation within 7 days, 4 were managed with NIV or HFNO due to post-extubation respiratory distress, and 5 died within 7 days of extubation. Some patients experienced more than one component of the composite endpoint.

### 3.1. Clinical Characteristics and Severity Indices

Patients in the weaning-failure group exhibited significantly greater illness severity at the time of extubation. The median Sequential Organ Failure Assessment (SOFA) score was higher among patients who failed weaning compared with those successfully weaned (7 [4–8] vs. 4 [2–6]; *p* = 0.006). Similarly, the Modified NUTRIC (mNUTRIC) score, reflecting systemic inflammation and nutritional risk, was elevated in the failure group (6 [5–7] vs. 4 [3–5]; *p* < 0.001). The Acute Physiology and Chronic Health Evaluation II (APACHE II) score was higher in the weaning-failure group (18 [11–28] vs. 12 [7–16]; *p* = 0.006). No significant intergroup differences were observed in age, sex distribution, BMI, frailty, or comorbidity burden as measured by the Charlson Comorbidity Index.

### 3.2. Laboratory Parameters

Biochemical assessment revealed a striking metabolic difference between groups.

The BUN/Creatinine ratio was markedly elevated in patients with weaning failure compared with those successfully weaned (58.7 [44.6–76.9] vs. 39.7 [23.8–49.2]; *p* = 0.007), whereas serum creatinine levels alone did not differ significantly (*p* = 0.41).

Serum albumin concentration was lower in the failure group (3.1 [2.8–3.4] vs. 3.5 [3.1–3.8]; *p* = 0.048), which may be consistent with greater metabolic stress and nutritional risk; however, renal and hemodynamic influences on BUN/Cr cannot be excluded.

There were no significant differences in total protein or inflammatory markers (CRP, leukocyte count) between groups.

### 3.3. Rectus Femoris Muscle Thickness and Sarcopenia

Bedside ultrasound evaluation demonstrated significantly reduced rectus femoris (RF) muscle thickness in the weaning-failure group (6.2 [5.4–7.0] mm) compared to the successfully weaned patients (7.8 [6.9–8.6] mm; *p* = 0.021).

When applying sex-specific thresholds (25th percentile of the cohort), ICU-acquired sarcopenia (ICU-AS) was identified in 40.5% of patients overall, and was significantly more prevalent among those with weaning failure (69.2% vs. 27.6%; *p* = 0.008).

**Figure 1:**
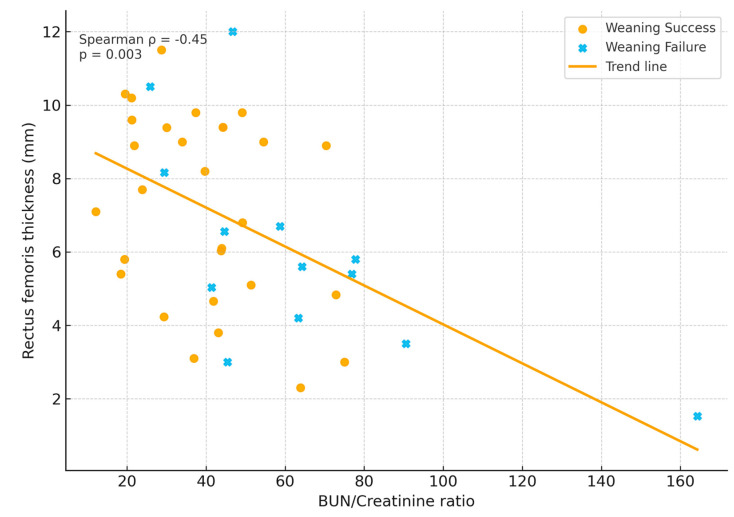
Relationship between BUN/Creatinine ratio and rectus femoris thickness.

Spearman correlation analysis revealed a moderate negative relationship between BUN/Cr ratio and RF thickness (ρ = −0.42; *p* = 0.008), consistent with the hypothesis that metabolic catabolism and structural muscle wasting progress in parallel (Figure 1).

### 3.4. Clinical Outcomes

The median ICU length of stay was 14 days [IQR 9–19], with no significant difference between groups (*p* = 0.61).

However, 28-day mortality was significantly higher among patients with weaning failure (53.8% vs. 6.9%; *p* = 0.002), underscoring the strong prognostic weight of failed ventilator liberation.

Patients requiring post-extubation noninvasive support or reintubation also exhibited higher baseline BUN/Cr ratios.

### 3.5. Multivariable Analysis

To evaluate independent determinants of weaning failure, a multivariable logistic regression model was constructed including BUN/Cr ratio, RF thickness, SOFA, mNUTRIC, and serum albumin (Table 2).

After adjustment, both metabolic and structural variables retained independent significance.

An elevated BUN/Cr ratio remained an independent predictor of weaning failure (OR 1.07; 95% CI 1.01–1.14; *p* = 0.024), whereas greater RF thickness was associated with a decreased risk (OR 0.78; 95% CI 0.62–0.98; *p* = 0.033).

Neither SOFA nor mNUTRIC remained significant after adjustment, likely reflecting limited power and collinearity among covariates.

### 3.6. Cut-Off Analysis and ROC Performance

Receiver operating characteristic (ROC) analysis identified a cohort-derived exploratory threshold of 44.6 for predicting weaning failure, corresponding to a sensitivity of 76.9% and specificity of 72.4% (AUC = 0.76, 95% CI 0.62–0.89).

**Figure 2:**
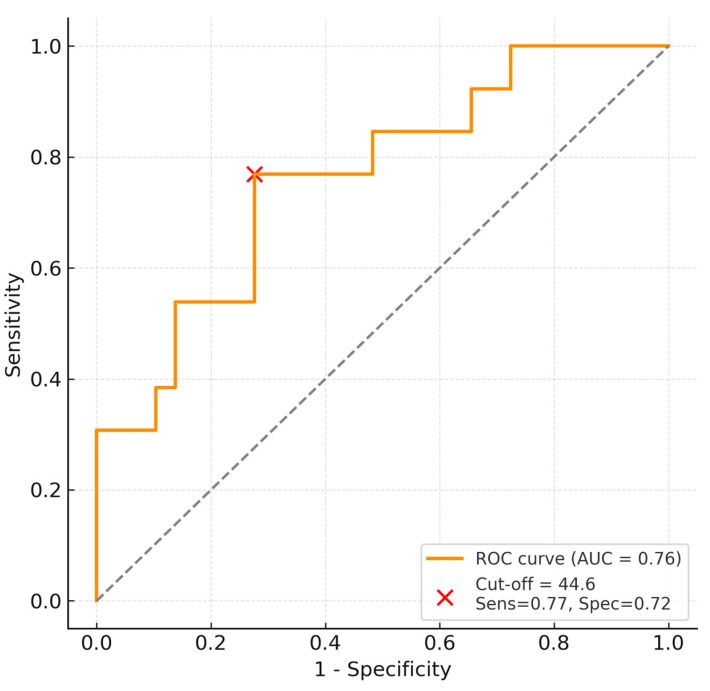
ROC curve showing predictive performance and optimal cut-off (44.6) for weaning failure. The dashed diagonal line represents the line of no discrimination (AUC = 0.5).

Patients exceeding this threshold were significantly more likely to experience weaning failure (Figure 2).

**Figure 3:**
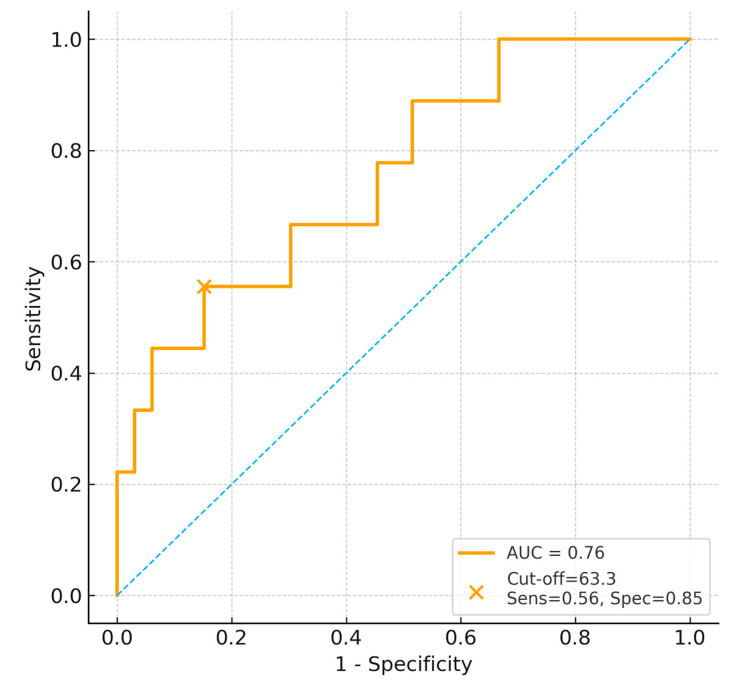
ROC curve for BUN/Creatinine ratio predicting 28-day mortality. The dashed diagonal line represents the line of no discrimination (AUC = 0.5).

In 28-day mortality; however, given the limited number of death events (*n* = 11), ROC-derived AUC estimates and cut-off values should be interpreted cautiously (Figure 3).

Rectus femoris thickness also demonstrated fair discrimination for weaning failure (AUC = 0.72), while the combination of metabolic and muscle parameters improved predictive accuracy (AUC = 0.81).

### 3.7. Composite Metabolic–Muscle Index

An exploratory composite indicator Metabolic–Muscle Index (MMI = BUN/Cr ÷ RF thickness) showed moderate discrimination in this cohort; however, it is ad hoc and unvalidated and should be considered hypothesis-generating.

**Figure 4:**
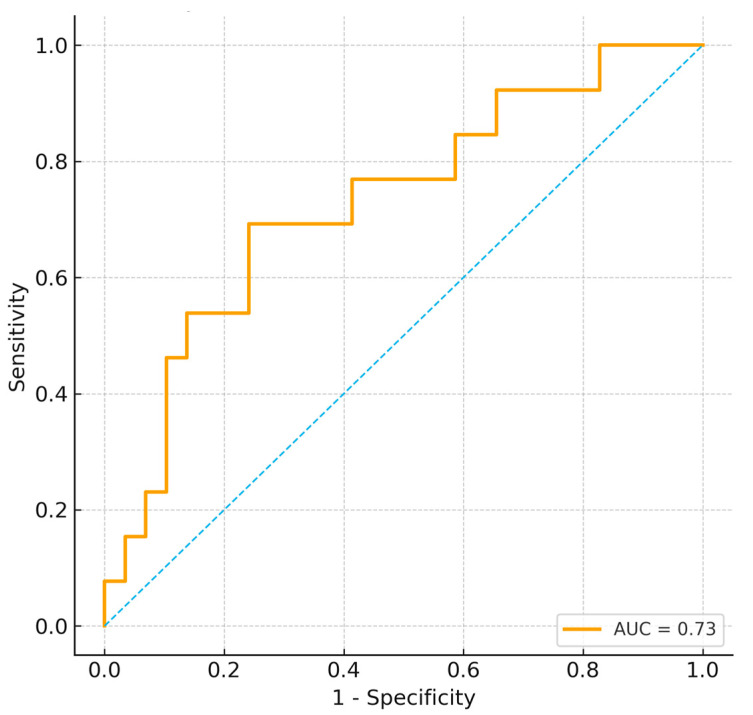
ROC curve for composite metabolic–muscle index (MMI) predicting weaning failure. The dashed diagonal line represents the line of no discrimination (AUC = 0.5).

Higher MMI values were strongly associated with weaning failure (*p* = 0.004), and ROC analysis demonstrated acceptable discrimination (AUC = 0.73; 95% CI 0.59–0.88) (Figure 4).

This composite measure may provide a pragmatic way to integrate metabolic and structural information for future validation studies.

### 3.8. Correlation Network

Further exploratory analysis revealed that the BUN/Cr ratio correlated positively with mNUTRIC (ρ = 0.41; *p* = 0.009) and negatively with albumin (ρ = −0.38; *p* = 0.011), while rectus femoris thickness correlated positively with albumin (ρ = 0.44; *p* = 0.006).

Together, these associations suggest an association pattern among metabolic, nutritional, and muscle-related variables.

## 4. Discussion

This study demonstrates that both elevated BUN/Creatinine ratio and reduced rectus femoris muscle thickness are associated with weaning failure and mortality; adjusted analyses suggested associations, but findings are exploratory given sample size and model limitations.

The findings provide biochemical and morphologic evidence that systemic catabolism and ICU-acquired sarcopenia jointly impair ventilator liberation.

### 4.1. Integration with Existing Literature

Our results corroborate the well-established link between muscle wasting and weaning outcomes.

Puthucheary et al. [3] first showed that critically ill patients lose up to 17% of rectus femoris cross-sectional area within one week of ICU stay, with consequent reductions in strength and functional recovery.

Subsequent studies by Dres et al. and Jung et al. confirmed that respiratory and limb muscle weakness are independent predictors of extubation failure [14,15].

In our cohort, patients with lower rectus femoris thickness experienced significantly more weaning failure, aligning with these findings.

The BUN/Cr ratio should be viewed as a nonspecific, integrative biomarker reflecting systemic metabolic stress, proteolysis, nutritional imbalance, and renal handling, rather than as a direct proxy for ICU-acquired sarcopenia. Its association with rectus femoris thickness suggests a link between biochemical catabolism and structural muscle loss, but does not imply causality.

Recent reports have highlighted its prognostic value in diverse critical illness contexts.

Paulus et al. [6] performed a 2025 meta-analysis identifying the urea-to-creatinine ratio as a marker of systemic catabolic activity, correlating with mortality, prolonged ICU stay, and muscle loss.

Similarly, Haines et al. [16] demonstrated that persistently elevated ratios after trauma reflect ongoing proteolysis and failure of anabolic recovery, even in the absence of renal dysfunction.

Jiang et al. [9] further linked BUN/Cr elevations with low skeletal muscle index on CT imaging in critically ill patients, suggesting that it mirrors muscle wasting on a biochemical level.

Our results extend these observations by confirming that the ratio predicts not only poor survival but also mechanical weaning performance—a clinically relevant endpoint.

Because no externally validated ultrasound cut-off values exist for defining ICU-acquired sarcopenia, we used a cohort-relative threshold to stratify patients by muscle thickness. This approach should be interpreted as an analytical tool to explore associations with clinical outcomes, rather than as a diagnostic classification of sarcopenia or functional impairment.

These findings illustrate the heterogeneous clinical trajectories encompassed by the composite definition of weaning failure, ranging from noninvasive respiratory support to reintubation and death.

Rectus femoris thickness was measured at a single, clinically meaningful time point (the spontaneous breathing trial) to reflect muscle reserve at the moment of ventilator liberation. This cross-sectional approach does not capture the dynamic trajectory of muscle wasting during critical illness.

The mortality analysis was based on a small number of events, which limits the stability of ROC estimates and precludes derivation of reliable thresholds. These findings should therefore be considered hypothesis-generating rather than definitive.

### 4.2. Pathophysiological Rationale

Traditionally, an increased BUN/Cr ratio was interpreted as a marker of prerenal azotemia.

However, in ICU patients, it may reflect the balance between urea generation and creatinine production—both of which are profoundly altered during catabolic stress [10].

Severe inflammation, corticosteroid exposure, and immobilization accelerate proteolysis, increasing hepatic ureagenesis and circulating BUN levels [11].

Concurrently, diminished muscle mass and reduced creatine turnover decrease creatinine production [17].

The result is a biochemical signature of hypercatabolism: increased BUN, stable or reduced creatinine, and an elevated BUN/Cr ratio.

Experimental data support this interpretation. Derde et al. [18] described a metabolic shift toward protein breakdown and suppressed anabolic signaling during critical illness, while Schefold et al. [19] emphasized that persistent negative nitrogen balance drives muscle atrophy and delayed weaning.

Longitudinal ICU studies have shown that rising plasma or urinary urea-to-creatinine ratios parallel losses in muscle cross-sectional area [7,10].

Therefore, BUN/Cr serves as a readily accessible marker linking biochemical catabolism with morphologic muscle wasting.

### 4.3. Combined Metabolic–Muscular Assessment

In our analysis, both BUN/Cr and RF thickness independently predicted weaning failure, and their combination (MMI) improved discriminatory power.

This finding underscores the complementary nature of biochemical and ultrasound-based monitoring.

While ultrasound visualizes structural atrophy, BUN/Cr quantifies metabolic imbalance driving that atrophy.

This integrated approach may refine patient phenotyping and enable early recognition of those requiring targeted interventions—such as optimized nutrition, early mobilization, and anabolic therapy [20].

### 4.4. Clinical Implications

BUN/Cr is inexpensive and routinely available, and rectus femoris ultrasound can be performed at the bedside. However, this study does not evaluate biomarker-guided weaning strategies. Therefore, proposed thresholds and combined assessments should be viewed as exploratory, and prospective multicenter studies are required to determine whether integrating these markers into weaning assessments improves clinical outcomes.

### 4.5. Comparison with Other Biomarkers

Various markers—such as serum albumin, prealbumin, and C-reactive protein—have been studied in the context of weaning outcomes.

However, these are often confounded by inflammation and hepatic function.

In contrast, the BUN/Cr ratio directly reflects nitrogen turnover, providing a more specific index of catabolic drive and substrate depletion.

Its association with muscle ultrasound findings supports its use as an objective, physiology-based biomarker of metabolic resilience.

The Metabolic–Muscle Index was conceived as a simple, illustrative composite indicator integrating biochemical and ultrasound-derived markers. Its mathematical form was not derived from mechanistic modeling or formal optimization, and it should not be interpreted as a validated risk score. Rather, it serves to highlight the potential value of multidimensional metabolic–muscular assessment in weaning research.

Although illness-severity and nutritional risk scores differed significantly between groups in univariate analyses, their lack of significance in multivariable models should not be interpreted as evidence of biological mediation. Given the limited number of events and potential collinearity with metabolic and muscle-related variables, these findings primarily reflect statistical constraints rather than mechanistic relationships.

Rather than reflecting a mechanistically specific pathway, the BUN/Cr ratio should be interpreted as a non-specific integrative biomarker capturing the combined effects of metabolic stress, renal physiology, nutritional status, and muscle substrate availability. Its association with rectus femoris thickness supports a link between biochemical and structural domains, but does not imply biological specificity.

## 5. Limitations

Several limitations should be acknowledged. First, the relatively small sample size and limited number of outcome events increase the risk of overfitting in multivariable analyses, particularly with respect to the events-per-variable ratio. Accordingly, adjusted results should be interpreted as exploratory and hypothesis-generating rather than definitive, and residual model instability cannot be excluded. Larger multicenter studies using parsimonious or penalized regression approaches are required to confirm these findings.

Renal function is an important potential confounder in interpreting the blood urea nitrogen–to–creatinine (BUN/Cr) ratio. Although patients with end-stage renal disease were excluded, acute kidney injury, hemodynamic fluctuations, diuretic exposure, and changes in renal perfusion may have influenced BUN and creatinine independently of muscle catabolism. Therefore, BUN/Cr should be considered a composite metabolic signal rather than a specific marker of sarcopenia.

ROC analyses and derived cut-off values were not internally validated, and model calibration was not formally assessed. Given the modest sample size, AUC estimates may be subject to optimism bias; thus, proposed thresholds are illustrative and not intended for clinical decision-making without further validation.

Low muscle mass was defined using a cohort-relative percentile rather than externally validated or functional criteria, which may classify a fixed proportion of patients by design. The proposed Metabolic–Muscle Index is an exploratory, ad hoc composite derived from the same dataset, lacking biological optimization and internal or external validation; its performance should therefore be regarded as hypothesis-generating.

Several relevant confounders influencing weaning outcomes were not systematically collected or adjusted for, including duration of mechanical ventilation before the spontaneous breathing trial, cumulative sedative or neuromuscular blocker exposure, fluid balance, cardiopulmonary comorbidities, and diaphragmatic function. Residual confounding cannot be excluded, and associations should not be interpreted as causal. Initiation of noninvasive ventilation or high-flow nasal oxygen may also reflect institutional practice patterns.

Ultrasound measurements were performed by a single operator without formal intra-rater reliability testing, and muscle thickness was assessed at a single time point, precluding evaluation of longitudinal muscle loss or differentiation between acute ICU-acquired wasting and pre-existing low muscle mass. Only 11 mortality events were observed, limiting the stability of mortality-related ROC estimates.

Published absolute cut-off values for rectus femoris thickness vary widely across studies, reflecting heterogeneity in patient populations, illness severity, timing of assessment, and ultrasound methodology. Mean rectus femoris thickness in our cohort was below several previously proposed thresholds; however, this does not imply universal sarcopenia, and highlights the limitations of applying absolute cut-offs across different ICU populations.

Finally, this retrospective single-center study may limit generalizability to other settings. Importantly, biomarker-guided weaning strategies were not evaluated, and no conclusions can be drawn regarding the impact of BUN/Cr or composite indices on clinical decision-making or patient outcomes.

## 6. Future Directions

Prospective multicenter studies incorporating serial BUN/Cr measurements, functional muscle assessment, and standardized weaning definitions are needed to validate these findings and determine whether dynamic changes in metabolic and muscle markers provide incremental information regarding readiness for extubation.

## 7. Conclusions

An elevated BUN/Creatinine ratio was associated with weaning failure and short-term mortality in mechanically ventilated patients, and correlated with lower rectus femoris thickness in this cohort. These findings suggest a conceptual association between biochemical and ultrasound-based structural markers during ventilator liberation; however, they should be interpreted as hypothesis-generating given the retrospective single-center design, limited event counts, and lack of internal/external validation. Future prospective multicenter studies are needed to determine whether integrating such markers into weaning assessments improves clinical outcomes.

## Figures and Tables

**Table 1:** Baseline characteristics and clinical outcomes according to weaning outcome.

Variable	All Patients (*n* = 42)	Weaning Success (*n* = 29)	Weaning Failure (*n* = 13)	*p* Value
Age, years, median (IQR)	73 (66–79)	72 (65–78)	75 (67–81)	0.258
Sex, male, *n* (%)	25 (59.5%)	17 (58.6%)	8 (61.5%)	0.41
SOFA score, median (IQR)	5 (3–8)	4 (2–6)	7 (4–8)	0.006
APACHE II score, median (IQR)	13 (9–20)	12 (7–16)	18 (11–28)	0.006
mNUTRIC score, median (IQR)	4 (3–6)	4 (3–5)	6 (5–7)	<0.001
Charlson Comorbidity Index, median (IQR)	6 (4–8)	6 (4–8)	6 (4–8)	0.53
BMI, mean ± SD	24.7 ± 4.2	24.6 ± 4.1	24.9 ± 4.3	0.43
Albumin (g/dL), median (IQR)	3.4 (3.0–3.7)	3.5 (3.1–3.8)	3.1 (2.8–3.4)	0.021
BUN/Creatinine ratio, median (IQR)	46.3 (32.9–61.8)	39.7 (23.8–49.2)	58.7 (44.6–76.9)	0.002
Rectus femoris thickness (mm), mean ± SD	6.63 ± 2.37	6.93 ± 2.31	5.96 ± 2.41	0.03
ICU length of stay, days, mean ± SD	14.9 ± 9.3	14.3 ± 8.8	16.1 ± 10.2	0.60
28-day mortality, *n* (%)	11 (26.2%)	2 (6.9%)	7 (53.8%)	0.005

Data are presented as median (interquartile range), mean ± standard deviation, or number (percentage) as appropriate. SOFA: Sequential Organ Failure Assessment; APACHE II: Acute Physiology and Chronic Health Evaluation II; mNUTRIC: Modified Nutrition Risk in Critically Ill; BMI: Body Mass Index.

**Table 2:** Multivariable logistic regression analysis for predictors of weaning failure.

Variable	Odds Ratio (OR)	95% Confidence Interval	*p* Value
BUN/Creatinine ratio	1.07	1.01–1.14	0.024
Rectus femoris thickness (mm)	0.78	0.62–0.98	0.033
SOFA score	1.12	0.89–1.41	0.298
Modified NUTRIC score	1.09	0.84–1.43	0.524
Albumin (g/dL)	0.74	0.43–1.26	0.259

Multivariable logistic regression analysis including BUN/Creatinine ratio, rectus femoris thickness, SOFA, Modified NUTRIC score, and albumin. BUN/Creatinine ratio and rectus femoris thickness were independent predictors of weaning failure.

## Data Availability

The datasets used and/or analyzed during the current study are available from the corresponding author on reasonable request.
